# Metabolic engineering of fatty acids from soybean seeds

**DOI:** 10.1186/1753-6561-8-S4-P93

**Published:** 2014-10-01

**Authors:** Alex Machado, André Murad, Giovanni Vianna, Nicolau Cunha, Cíntia Coelho, Marly Coelho, Elibio Rech

**Affiliations:** 1 University of Brasilia, Brasilia, Brazil; 2 Embrapa Genetic Resources and Biotechnology, Brasilia, Brasil

## Background

The worldwide demand for new renewable sources of energy is rising every day [1]. Biodiesel is a green fuel made mainly from soybean oil, on average composed of 25% oleic acid and 13% palmitic acid, which negatively impacts its oxidative stability and freezing point, causing a high rate of nitrogen oxide emission [2,3]. To improve the quality and performance of soybean oil as biodiesel, manipulation of its production of fatty acids is required to increase monounsaturated acids and reduce polyunsaturated acids. This work focuses on metabolic engineering of soybean to achieve those requirements.

## Methods

The genes *FAD2-1 *and *FatB*, encoding Δ12 desaturase and a palmitoyl thioesterase respectively, are primarily responsible for regulating the production of oleic and palmitic acids. The soybean genes *FAD2-1 *and *FatB *were placed under the control of constitutive promoter 35SCaMV, introduced to soybean embryonic axes by particle bombardment [4] and down-regulated using RNA interference technology. Plants were grown in greenhouse and PCR was performed to isolate potential genetically modified soybean. Oil extracted from seeds obtained from positive PCR plants was analysed by gas chromatography mass spectrometry using a modified protocol from Primomo *et al*. [5].

## Results and conclusions

1000 embryonic axes were used in bombardment, 10 plants were recovered from herbicide selection and only 1 contained the RNAi transgene pattern. The metabolically engineered plant exhibited a significant increase in oleic acid (up to 94.58%) and a reduction in palmitic acid (to <3%) in its seed oil content. The soybean fatty acid composition was characterised by GC-MS. No structural differences were observed between the fatty acids of the transgenic and non-transgenic oil extracts. We describe the production of fatty acids in metabolically engineered soybean seeds in which high levels of oleic acid accumulated and the levels of palmitic acid were reduced. This lineage can be used for the production of high quality biodiesel.
